# A qualitative study on rural women’s experiences relating to the utilisation of birth care provided by skilled birth attendants in the rural areas of Bongo District in the Upper East Region of Ghana

**DOI:** 10.1186/s12884-019-2337-0

**Published:** 2019-06-06

**Authors:** Peter Adatara, Johanita Strumpher, Esmeralda Ricks

**Affiliations:** 1 Department of Nursing, University of Health and Allied Sciences, PMB 31, Ho, Ghana; 2 Department of Nursing, Nelson Mandela University, P O Box 77000, Port Elizabeth, 6013 South Africa

**Keywords:** Labouring women, Experiences, Utilisation, Skilled birth care, Skilled birth attendants, Rural areas

## Abstract

**Background:**

Increasing skilled attendance during childbirth is well established in literature to play a significant role in averting the many preventable maternal deaths that occur in developing countries such as Ghana. Inadequate utilisation of skilled birth care services in Ghana is believed to be a major hindrance to efforts aimed at improving the health of women, especially during delivery. The purpose of this study was to explore and describe the experiences of rural women regarding the utilisation of skilled birth care provided in the rural areas of Northern Ghana.

**Methods:**

The study adopted a qualitative, exploratory, descriptive research approach, whereby individual interviews, observations and field notes were used to obtain a thick description of women’s experiences regarding the utilisation of skilled birth care services in the rural areas in Ghana. A purposive sampling approach was used to select 20 women who utilised skilled birth care in the rural areas of Bongo District of Ghana. Data collected from the interviews were transcribed verbatim and analysed to identify themes.

**Results:**

The study identified three themes: The women had negative interactions with skilled birth attendants during delivery; women lacked confidence in skilled birth attendants’ abilities; skilled birth attendants disrespected the traditional beliefs of the women.

**Conclusion:**

Most of the participants had negative experiences regarding the utilisation of skilled birth care. There is thus a need to develop strategies that could help address the above concerns of women to facilitate utilisation of skilled birth services in the rural areas in Ghana where there is low utilisation of skilled birth care.

## Background

One of the strategies identified by the World Health Organisation as being crucial for saving the lives of pregnant women and as an indicator for progress in the reduction of maternal mortality is provision and utilisation of skilled birth care [1]. Skilled birth care describes a care by which a woman is provided with adequate care during labour, delivery and the immediate postpartum period by a trained health care professional [1]. Skilled care at birth occurs in a health facility or homebirth setting, assisted by trained professionals including midwives, nurses and doctors.

Ghana as a middle-income country within the Sub-Saharan African region although the skilled birth attendance has improved over the years, more than 20% of women still give birth at home without the assistance of a skilled birth attendant [2]. In rural settings in Ghana, more than 30% of women deliver at home without the assistance of a trained health care professional [2]. It is reported by the World Health Organisation that inadequate utilisation of skilled birth care services in Sub-Saharan Africa is a major hindrance to efforts aimed at improving the health of women, especially during delivery [3]. Adequate and competent maternal health care is a top priority for health intervention in Ghana, yet the researchers observed that few women seek assistance from trained health care professionals.

In Sub-Saharan Africa, many women deliver their babies at home in rural areas and are usually attended by various untrained family members, or the woman delivers alone unattended [4]. In Ghana, statistics from the Ghana Statistical Service (GSS) revealed that the percentage of births occurring in health facilities has increased from 57% in 2012 to 73% in 2014 [5]. A significant disparity exists between the utilisation of skilled birth care services in urban and rural areas in Ghana. The utilisation of skilled birth care is even lower in some parts of the country, especially the three Northern regions of Ghana [6].The Northern regions have consistently performed poorly in skilled birth attendance at delivery from 1990 to 2012 [5]. The exposure of women to poor care during labour and birth at home and by untrained TBAs, poses a great danger to the health of women [6].

Previous studies indicate that, lack of financial or economic resources, transportation, and delivery of supplies, lack of coordination and referral between traditional birth attendants at the community level and facilities can all inhibit women from using facility-based services [5–10]. However, the majority of these studies have been urban-focused, and consequently, rural women’s perspectives have been less discussed or studied. In addition, most of the studies have adopted quantitative approaches which limit a deeper understanding of women’s experiences regarding the use of skilled care during childbirth in the northern part of Ghana where there is underutilisation of skilled birth care services. This lack of attention is particularly true in the northern part of the country, where women are confronted with diverse barriers such as poor quality of maternal health care services, poverty, difficulties in transportation and traditional beliefs surrounding childbirth in the Bongo district in the Upper East Region of Ghana [11, 12].

In spite of several efforts by the government and other stakeholders such as the introduction of free maternal healthcare policy to facilitate the use of skilled birth care in Ghana, low utilisation of maternal health services especially making use of skilled birth care still persists in the rural areas of Bongo District in Northern Ghana. The researchers observed that women, especially those from rural and deprived communities in the Bongo District of Ghana continue to deliver at home without the assistance of skilled birth attendants. The purpose of this study is to describe women’s experiences regarding the utilisation of skilled birth care in the Bongo district in the Upper East Region of Ghana.

## Materials and methods

### Research design

A qualitative, explorative and descriptive research approach was employed whereby individual interviews, observations and field notes were used to obtain a thick description of women’s experiences regarding the utilisation of skilled birth care services in the rural areas in Ghana. Qualitative explorative and descriptive research is focused on understanding human’s experiences as it is lived, usually through the careful collection and analysis of qualitative materials that are narrative in nature [12]. This design enabled the researchers to gain an in-depth understanding of rural women’s experiences with regard to utilising skilled birth or unskilled birth services from their own perspective.

### Research setting

The research was carried out in the Bongo District in the Upper East Region of Ghana. The Bongo District was selected as the setting for conducting the study because Bongo District is one of the most rural and deprived districts in Ghana and has all the characteristics of a typical rural area in Ghana [13]. Also, the Bongo district was chosen because the district is one of the districts in Ghana with low utilisation of skilled birth care provided by skilled birth attendants. Ninety four percent (94%) of the population in the Bongo district reside in the rural areas [13]. The Bongo District is one of the six districts in the Upper East Region of Ghana, with Bongo Township as its district capital.

The health care delivery system is built around one district hospital in the district capital serviced by four reproductive Health Clinics, seven completed Community Health-based planning Services (CHPS) compounds, 62 outreach points, 10 feeding centres and one rehabilitation centre. According to Bongo District Health Directorate there is one doctor in the district, the District Medical Superintendent who oversees the District Hospital and 65 nurses in the entire district [14]. In the Bongo district, with exception of only one district hospital which has one general practitioner (medical doctor) and midwives providing skilled birth care, the rest of the maternity units in health facilities in the district are manned by midwives providing primary maternity care for women during pregnancy and childbirth. There are a variety of alternative childbirth sources available in these communities; they include TBAs, traditional healers and herbalists, spiritual healers and diviners.

### Research population and sampling

The research population for this study comprised of twenty (20) women who gave birth within 6 months in a health facility. To understand women’s experiences regarding the utilisation of skilled birth care services from their own perspective, a purposive sampling technique was utilized to select 20 participants who gave birth within 6 months period in a health facility.

Bongo District is sub-divided into six sub-districts or zones according to Bongo District Health Directorate. Two Zones were randomly selected for the study. These two zones were believed to have performed poorly in skilled birth care attendance in the Bongo District over the years prior to data collection. Nurses and Community Key Informants (CKIs) provided a list of potential respondents (women) who delivered within 6 months utilising skilled birth care in each of the selected zones who were willing to participate in the study and met the inclusion criteria after the purpose of the study was explained to them. In order to maximize the variability of the sample and get diverse experiences, participants were purposively selected taking into consideration respondents’ age, parity, educational status, employment status, marital status and type of delivery, that is whether delivery was vaginal, assisted or caesarean section.

### Inclusion criteria

- Mothers who delivered live babies in health facilities assisted by skilled attendants within 6 months’ period and whose babies at the time of the study were still alive and well.
- Women who were above 18 years of age

### Data collection process

Data was collected by means of a semi-structured interview approach, which afforded the researchers flexibility. Prior to beginning data collection, the purpose of the interview was explained to participants. Confidentiality was assured and informed consent was obtained. The consent form was read in the Grune, a language spoken in the study area to participants who could not read or write and provided a thumbprint. Participants were informed they could decline to answer any question or stop talking at any time they wished during the interview process for any reason. They were also informed they could request the audio-recorder be turned off at any time.

A semi-structured interview approach was utilised to gain a full understanding of women’s experiences regarding the utilisation of skilled birth care services. The registered midwives and community health nurses working at the antenatal and child welfare clinics were the gatekeepers as they assisted the researchers in choosing 20 participants from amongst the sample group of 110 women attending the child welfare clinics in two zones that had low levels of skilled birth attendance were selected for the study. Interviews were conducted by one of the researchers who worked in the study area for 5 years and could speak the Grune language fluently. The interviews took place at the community social centres perceived as convenient and comfortable and in a Grune language, preferred by participants. The participants suggested the community social centres in the communities as the most preferred venues for the individuals because those venues were free from interruptions. A voice recorder was used to capture data during sessions. Each interview took between 40 to 60 min. The audio-taped interviews were transcribed within 24 h of the interview while the information was still fresh in the researchers’ mind. A language expert translated the interviews into the English Language to enable the promoters of the study and the independent coder to understand the content of the interviews. The transcribed interviews formed the database of the study. The same questions were posed to all participants in the preferred language which was Grune. The following questions were posed to women who utilized a skilled birth care:

- Please, tell me all about your experiences during the antenatal care of this current baby?
- Please tell me all about your experiences during the labour and birth of this current baby?
- Please tell me what informed your decision to choose your place of birth?
- Please tell me what kind of childbirth care you would have liked to have had at where you gave birth to your child?

### Data analysis

Data collected from the semi-structured interviews were transcribed verbatim and analysed according to the steps suggested by Tesch, to identify themes and sub-themes relevant to the development of case studies [15]. The approach involves breaking down narrative data into smaller units, coding and naming the units according to the content they represent and grouping coded material based on shared concepts and meaning [16]. Interviews were transcribed, read and reread to identify the important themes and categories and sorted to sub-themes to reconstruct a description of how cultural beliefs and practices influence the choice of place for childbirth. Transcripts were read several times to develop a coding framework, which was finalised through consensus between co-authors. Also, transcripts from the audio-taped interviews were made and were sent to an independent coder who has experience in qualitative data analysis at the PhD level, together with a data analysis guide. The use of an independent coder was to assist in the exclusion of biases by the researchers and to control potential haphazardness with the data analysis. The interviews were analysed according to the protocol used by the researchers. The independent coder was instructed to use the data analysis guide provided to analyse data from the transcribed interviews to assist in excluding biases of the researcher. The data analysis occurred independently, and the researcher and the independent coder met after the completion of the individual analysis for consensus discussions. These discussions assisted in reducing the data to categories and sub-categories and main emergent themes.

### Ethical considerations

The research had ethical approval from the Faculty Post Graduate Studies Committee at Nelson Mandela University **(**H14-HEA-NUR-30**).** The researcher also requested permission in writing from the Bongo District Assembly and the Bongo District Directorate of Health Services to conduct research in the communities in the Bongo District. The researcher then requested permission from the community opinion leaders namely, the chiefs and the assemblymen, who were approached by means of a formal written request so that they become personally informed about the researchers presence in the communities under their direct jurisdiction. Informed consent was obtained from the research participants by issuing each participant with a letter that explained to them in full what the purpose and objectives of the research study were. Translation and interpretation of the information concerning the purpose and objectives into the Grune language was done for those who could not read in the English language. Each participant was given time to read the letter. The consent forms were signed by each participant who could read, and thumb printed by participants who could not read.

## Results

### Characteristics of the participants

The characteristics of the participants show 95 % (*n* = 19) of the participants, belonged to the Frafra ethnic group whilst one (5%) participant belonged to the Builsa ethnic group. Twelve (60%) of the participants were married, six (30%) were single parents and two (10%) were widowed. The parity of participants ranged from one to six children. The youngest participant was 18 years and the oldest was 40 years. The majority (35%, *n* = 7) of the participants were within the age range of 15–20 years. The majority (80%, *n* = 16) of the women who participated in the study had no formal education. Only 20% (*n* = 4) of the participants had formal education up to primary or Junior High School level or dropped out at various stages of Junior High School. Also, the majority (85%, *n* = 17) of the participants were engaged in farming activities of various kinds to earn a living.

### Identified themes

Three themes emerged from the data analysis:

- The women had negative interactions with skilled birth attendants during delivery
- During delivery women lacked confidence in skilled birth attendants’ abilities
- Skilled birth attendants disrespected the traditional beliefs of the women

The participants based their opinions regarding the use of skilled birth care on their experiences of childbirth. The experiences of women during childbirth are an important determinant in the utilisation of skilled birth care.

#### Theme 1: the women had negative interactions with skilled birth attendants during delivery

The women indicated they experienced negative interactions with skilled birth attendants during childbirth. Interviews with the women indicated that they had a negative interaction with skilled birth attendants during birth. One of the participants expressed her concerns regarding the negative interaction she had with skilled birth attendants when she went to give birth in the health facility.

“…. when I got there and gave the referral note to the midwife on duty, and she read it, she wouldn’t even ask me what really happened, but she started raining insults on me and said why was it that all that the midwife asked me to do I refused to do. I kept quiet because she wouldn’t allow me to explain what happened and I was referred to them. So, after insulting me and saying all those things, she took me inside but then all the accusations levelled against me were not true”.

The participants also indicated although that they had a positive interaction with the mature and the older midwives, their interactions with the younger midwives were bad. The participants however revealed that in order to receive the care and treatment they needed or avoid getting into conflicts with nurses; they often have to accept the scolding, intimidation and disrespect in maternity wards without openly expressing their feelings. A participant shared the following experience:

“The elderly midwives were so nice to me because they were always talking to me and my mother and advising me. Even when I was pushing one of them was always praising me by saying, ‘oh you are doing well, good girl,’ and I think that actually motivated me to push further and to endure the pain. But then not all of them were good. I believe if one of the elderly midwives were around the day I was struggling to deliver by myself, I wouldn’t have suffered that way”.

Related to negative interaction with skilled birth attendants, some of the participants described skilled birth attendants not only being uncourteous but also verbally and physically abusive, rude, bossy, disrespectful, insulting, easily angered, having poor attitudes, and lacking compassion which did not help them during their childbirth. This problem is captured in the quote below:

“The nurse told me that I was a witch because when she slapped me and went home to sleep, she dreamt that she had a misunderstanding with me and I beat her up and because of that she thought I was a witch, and that I should leave her alone. But her insults drew people’s attention to me, and when the people asked me why we were talking, I explained to them and everyone started laughing, but they asked me not to mind her because she was guilty of what she did to me the previous day”.

#### Theme 2: during childbirth women lacked confidence in skilled birth attendants’ abilities

The participants reported they lacked confidence in skilled birth attendants’ abilities to conduct safe childbirth. Women’s confidence in skilled birth attendants’ abilities to conduct labour and childbirth safely and successfully is a determining factor in the utilisation of skilled birth care. The participants indicated that they experienced certain attitudes and behaviours from skilled birth attendants during their recent childbirth, making them doubt their competency and ability. The lack of confidence in skilled birth attendants were expressed by women as follows:

“As for some of those midwives they don’t know anything. They behave as if they have not been trained to conduct labour and childbirths”).

“Some of the nurses these days are just there for the money, but they don’t know the job. When you are in labour and you go to give birth, sometimes they would just be struggling with you without knowing what they are doing and if you are lucky they will eventually manage to help you give birth”.

The women also indicated that they had no trust in the abilities of the younger midwives and nurses. Some of them indicated that, although the young midwives do not know their work well, they are the ones who usually misbehave towards women in labour. This is evident by a quote from a participant below:

“I have realised that it is the small, small girls (referring to young midwives) who don’t even know the work well, and yet they don’t respect us at all. For instance, in my own case that I narrated to you about my previous delivery at Bongo Hospital, the nurse examined me and told me I was not due for labour, so I should go back and be roaming, but that was not the true picture about the stage of the labour. I eventually delivered less than 30 minutes”.

Additionally, some of the participants indicated that some of the skilled birth attendants were unable to recognize true labour signs. Participants did not have the needed confidence in the abilities of skilled birth attendants because they are not able to identify where they were in labour and went to the health facility to give birth. The participants expressed concerns that the inability to recognize labour has often resulted in poor birth outcome for the mother and the child as indicated below:

“…I must say that some of the nurses are unable to recognise the signs of labour because when you are in labour and go them they are unable to tell whether you are due to give birth or not…”.

“...As for some of the nurses they can’t recognise labour and are not sometimes able tell you whether what you are experiencing is as a result of true labour or not”.

The participants also felt that the skilled birth attendants were supposed to provide them with the right information regarding the labour process but they failed to provide or teach them but expected them to know the process. This issue is highlighted in the statement below:

“The midwives wouldn’t just tell us what to do at each stage of the labour especially those of us delivering for the first time, so that you are able to follow their instructions and yet when you don’t follow their instructions during labour, they would speak to you as if we are trained about the delivering process. Communication is what is always creating the confusing between us the women and the nurses during childbirth”.

#### Theme 3: skilled birth attendants disrespected the traditional beliefs of the women

Disrespect for the traditional beliefs of the women was one of the experiences of women’s utilisation of skilled birth care in the study area. Women reported significant disrespect of their traditional beliefs during childbirth by skilled birth care attendants in the health facilities, even though those cultural or traditional beliefs are not harmful to the life of the mother and the unborn baby.

The participants in this study indicated that certain cultural practices such as taking the placenta home by relatives or mothers to be buried according to their tradition were denied by skilled birth attendants after childbirth and instead, decided to dispose thereof according to the hospital protocol and policies. The sentiment of one of the women was expressed below:

“When I delivered at the hospital, my mother requested for the placenta to be taken home for burial according to my tradition, but the nurse refused to give it to her. My mother even pleaded with the nurse for about 30 minutes and she still refused to give it to us”.

“One thing that annoyed me and my family was the refusal by the nurses to allow us to take the placenta home for burial”.

The participants indicated that they perceived that midwives refused to release the placenta to family members because they wanted to burn the placenta which was unacceptable. They indicated that such practice of burning the placenta could have dire consequences on the destiny of the child. The participants indicated that the placenta is usually supposed to be buried outside the house at a place called “Tampugre” and not to be burnt.

“The health personnel refused to give the placenta to us to take home but rather took it themselves and went and burnt it. I don’t know where they burn them in the hospital, but I am told they always burn it. But you see burning the child’s placenta, means you’re burning his destiny for life”.

The participants in this study reported that some of the skilled birth attendants refused to allow family relatives or mothers give certain food they believed to be culturally accepted according to the tradition of women. Women reported that normally when a woman gives birth newly, the first thing they give to her according to their tradition was hot “*zoomkom”* (a local drink prepared from millet) or “*puusakoom”* (a local drink prepared from fruits). A participant expressed her sentiment in the quote below:

“When I gave birth at Namoo clinic my mother brought me “Zoomkom” but the midwife refused to allow me drinks it. She rather asked my mother to go and buy tea for me. But, I couldn’t take it well because I didn’t have an appetite for a tea. I wanted zoomkom or pusakoom”.

“I don’t understand why the midwives wouldn’t always allow us to take our zoomkom or puusakoom after giving birth, but they would rather recommend a tea for us as if as we are white people”.

## Discussion

The aim of this study was to explore and describe rural women’s experiences in the utilisation of skilled birth care. The findings of this study show that labouring women in rural areas in the Bongo District of Ghana had negative experiences utilising skilled birth care. The participants based their opinions regarding the use of skilled birth care on their experiences of childbirth.

The results of this study show that most of the women attended the antenatal clinic when they were pregnant and that influenced some of women decision to give birth in a healthcare facility. The findings of this study confirm a report from the 2014 Ghana Demographic and Health Survey (GDHS) which shows that due to women’s awareness of the importance of ANC, 87% of pregnant women in Ghana attended ANC at least one to four times prior to giving birth [17].

Also, the findings of this study revealed that some of the participants in this study had negative interactions with skilled birth attendants during childbirths. Interviews with women indicated that they had negative interactions with skilled birth attendants during childbirths. Participants reported that skilled birth attendants such as nurses and midwives used abusive language against women who utilised health facilities for childbirth in the study area. The attitudes of some of the midwives have made some of the women to regret having taken a decision to give birth utilising the services of skilled birth attendants. Similarly, it is reported in literature that an important factor expectant mothers consider in utilising skilled birth care is the quality of the patient-provider interaction and communication [18]. In a recent qualitative study conducted to explore the facilitators and barriers to facility-based delivery in low- and middle-income countries, it was reported that women experienced verbally abusive words and rude behaviors from skilled birth attendants during childbirth [19]. These negative childbirth experiences of women utilsing skilled birth care in this study may probably be due to the fact that majority of the participants in this study were from the same ethnic group which might have influenced their experience. Notwithstanding, the results of this study imply there is need for midwives to improve their interpersonal interaction with the labouring women in order to facilitate the utilisation of services.

Furthermore, this study found that during childbirth women did not trust the confidence of skilled birth attendants’ abilities. It was reported by most of the participants during the interviews that they did not trust the abilities of the skilled birth attendants especially the younger ones. Women’s confidence in skilled birth attendants’ abilities to conduct childbirth safely and successfully is a determining factor in the utilisation of skilled birth care. Previous researchers have reported similar findings that some women did not have confidence in the abilities of skilled birth attendants which usually influences birthplace decision-making of mothers [20–22]. Young skilled birth attendants such as nurses and midwives might feel aggrieved by these accusations because of the fact they are still new in practice and are still learning how to master their knowledge and skills to win the trust and confidence of labouring women. Notwithstanding, those in charge of maternity wards should ensure that inexperience midwives are not left alone to care for women in labour without the support of the experienced midwives.

Additionally, lack of cultural sensitivity or respect for women’s traditional beliefs was one of the experiences of women’s utilisation of skilled birth care in the study area. Some of women reported significant lack of cultural sensitivity or respect for traditional beliefs such as not allowing relatives to take home the placenta to be buried according to their tradition, not allowing certain foods, birthing position as well allowing their mothers-in-laws to stay with labouring women during childbirth by skilled birth attendants in the health facilities, even though those cultural or traditional beliefs are not harmful to the life of the mother and the unborn baby. In general, although giving the placenta to women or their family members to take home has no health implication, midwives do not usually give the placenta to the woman or her family members after childbirth to take home but are rather disposed of by themselves with the reason it was against the facility policy to give placenta to a woman’s relatives to take home. There have been several calls in Ghana, urging skilled birth attendants to integrate certain cultural practices that are held in high esteem by women and do not cause any problem to the expectant mother and unborn baby into the healthcare delivery system, to promote the utilisation of skilled birth care [18]. Despite the several calls made to skilled birth attendants to respect women’s traditional beliefs and values during childbirth, interviews with women showed that certain traditional beliefs concerning childbirth are of special value to women in Ghana and therefore, need to be respected by skilled birth attendants.

Although this study provides important insights to experiences of women utilising skilled birth care provided by skilled birth attendants in the rural areas of Bongo district in the Upper East of Ghana, there are some limitations that need to be acknowledged. This study selected only women who gave birth utilising skilled birth care services. The inclusion of women who gave birth without utilising skilled birth care in the study would have enriched the findings of this study. It is therefore recommended that further studies be conducted to explore the experiences of both mothers who utilised or did not utilised skilled birth care during birth in order to compare their experiences. Also, it was observed that the majority of the participants in this study were from the same ethnic group and that might have influenced mothers experience in utilising the skilled birth care. Notwithstanding the limitations highlighted above this study achieved its objective of exploring and describing the experiences of women utilising birth care provided skilled birth attendants in in the rural areas of Bongo district in the Upper East of Ghana.

## Conclusion

This study gives a unique insight into women’s experiences of utilisation of the services provided by skilled birth attendants. The findings of this showed that most the women who utilised skilled birth care during childbirth in study had negative experiences as women experienced verbally abusive words and rude behaviors and lack of cultural sensitivity or respect for traditional beliefs by skilled birth attendants. This study also showed that some women did not have confidence in the abilities of skilled birth attendants to provide safe childbirth care. There is the need for maternal healthcare managers to address the poor attitudes and lack of cultural sensitivity or respect for traditional beliefs by skilled birth attendants to facilitate utilisation of the services provided by skilled birth attendants in the rural areas in Ghana where there is low utilisation of skilled birth care.

### Recommendations for nursing and midwifery practice

- The results of this study point to the fact that there is the need for healthcare managers in rural communities in Ghana to develop policies and strategies that will make skilled birth attendants such as nurses and midwives in rural areas to be culturally sensitive to the cultural beliefs and practices of women during labour and birth in the health facilities.
- Newly qualified nurses and midwives should be given orientation training as well as supportive supervision and mentoring by senior and experienced midwives to enhance their knowledge and skills in childbirth care
- The Nursing and Midwifery Council of Ghana should organise specialized short courses covering all areas of maternity care to update the knowledge and competencies of maternity care providers. It should be made compulsory for all midwifery practitioners to attend.

### Recommendations for nursing research

A further research study could be conducted to assess stakeholders’ perspectives on the barriers to skilled birth care in both rural and urban communities in Ghana to enable the development of good strategies and policies that could be used by skilled birth care providers to facilitate the use of skilled birth care by women. A research study should be conducted on how to integrate traditional birth attendants into the orthodox health care practice.

## Abbreviations

CHPS: Community Health-based planning Services
CKIs: Community Key Informants
GDHS: Ghana Demographic and Health Survey
GSS: Ghana Statistical Service
TBA: Traditional Birth Attendant
